# Seconds-Resolved Measurements of Vancomycin Transport from the Plasma to the Interstitial Fluid Highlight a Path Towards Real-Time Therapeutic Drug Monitoring

**DOI:** 10.3390/s26072233

**Published:** 2026-04-04

**Authors:** Julian Gerson, Murat Kaan Erdal, Lisa C. Fetter, Kaylyn K. Leung, Nicole A. Emmons, Joao Hespanha, Carl M. Kirkpatrick, Kevin W. Plaxco, Tod E. Kippin

**Affiliations:** 1 Department of Psychological and Brain Sciences, University of California, Santa Barbara, Santa Barbara, CA 93106, USA; 2 Neuroscience Research Institute, University of California, Santa Barbara, Santa Barbara, CA 93106, USA; 3 Department of Bioengineering, University of California, Santa Barbara, Santa Barbara, CA 93106, USA; 4 Department of Electrical and Computer Engineering, University of California, Santa Barbara, Santa Barbara, CA 93106, USA; 5 Biomolecular Sciences and Engineering, University of California, Santa Barbara, Santa Barbara, CA 93106, USA; lisafetter@ucsb.edu; 6 Department of Molecular Cellular and Developmental Biology, University of California, Santa Barbara, Santa Barbara, CA 93106, USA; 7 Monash Institute for Pharmaceutical Sciences, Monash University, Melbourne 3800, Australia; carl.kirkpatrick@monash.edu; 8 Department of Chemistry and Biochemistry, University of California, Santa Barbara, Santa Barbara, CA 93106, USA

**Keywords:** biosensor, dermis, peripheral tissue, pharmacometrics

## Abstract

Continuous, in vivo drug and biomarker measurements could transform healthcare, enabling both the high-precision personalization of drug dosing and the real-time monitoring of health status. A practical realization of this vision, however, requires an improved understanding of the relationship between concentrations measured in the easily accessible dermal interstitial fluid (ISF) that correlate with the plasma concentrations that guide clinical decision making. As a preliminary step towards this goal, here we have used electrochemical, aptamer-based (EAB) sensors to perform seconds-resolved vancomycin measurements in the plasma and subcutaneous ISF of live rats. Concentrations of the antibiotic in the ISF vary rather little between different subcutaneous sites and, after the very rapid initial distribution phase is complete, they are well correlated with the plasma concentrations (mean R^2^ = 0.88). Likewise, a simple, two-compartment, two-parameter model describes our six paired plasma and ISF drug time courses quantitatively. Together, these findings provide further evidence of the viability of the drug concentration measurements performed in the subcutaneous or dermal ISF as a less invasive approach to real-time drug monitoring in individual patients.

## 1. Introduction

The ability to measure the concentrations of drugs and biomarkers in the body continuously and in real time would greatly improve our ability to diagnose, monitor, and treat disease. For example, by providing real-time information regarding blood sugar, the continuous glucose monitor (CGM) has revolutionized the treatment of diabetes [1,2]. The expansion of such real-time, in vivo monitoring to other molecules could prove similarly important. The monitoring of creatinine, for example, would provide a real-time window into kidney function, speeding up the detection—and thus treatment—of acute kidney injury. Likewise, the real-time monitoring of plasma drug concentrations would enable truly personalized dosing, in which low accuracy predictions based on one or two laboratory-derived drug concentration measurements are replaced by high-precision, several-thousand-timepoint measurements of each individual patient’s pharmacokinetics. Such real-time drug measurements could even be used to perform closed-loop feedback-controlled drug delivery, an ultra-high-precision dosing approach by which drugs with narrow therapeutic windows or complex optimal dosing regimens could be delivered safely and effectively [3,4,5]. Two advances, however, must be achieved before the in vivo measurement of drugs: biomarkers and other molecules will become as convenient as the continuous glucose monitor has rendered the measurement of blood sugar. First, the realization of such a vision requires the development of a “platform” technology that, unlike the glucose monitor, can be generalized to the detection of a wide range of molecules in the body. Second, it requires a quantitative understanding of how pharmacokinetics in the easily accessible, subcutaneous interstitial fluid (ISF) correlates with the plasma pharmacokinetics that are the basis of all essential clinical decision making.

**Figure 1:**
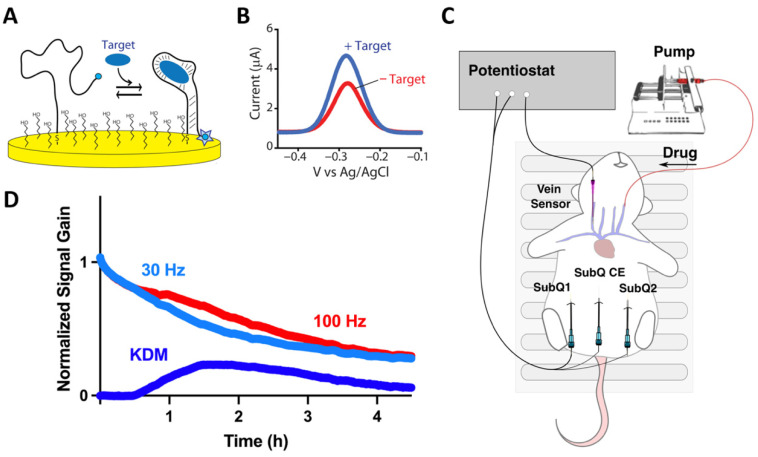
(**A**) EAB sensors comprise an unfolded, target-recognizing aptamer that has been site-specifically attached to an electrode and modified with a methylene blue redox reporter. Binding-induced folding of the aptamer alters the rate of electron transfer from the reporter, producing (**B**) an easily measurable signal when the sensor is interrogated, using square wave voltammetry. (**C**) Here, we have employed EAB sensors to measure the kinetics of drug disposition in plasma (in the jugular) and in the subcutaneous interstitial fluid (ISF). For the latter measurements, one or two sensors were placed in the subcutaneous space (SubQ1/2) of the animal’s ventral surface, with the necessary counter electrode (CE) being placed in the subcutaneous space a short distance away. (**D**) Critical to achieving in vivo measurements is the ability to correct for the drift seen in in vivo sensor placements. To achieve this, we use kinetic differential measurements (KDM), which employ signals collected at a pair of square wave frequencies that drift in concert but respond differentially to target binding.

Continuous glucose monitors rely critically on the enzymatic conversion of their target into an electroactive product, a highly specific chemical reaction that is not transferable to the detection of most other clinically important molecules. For example, while some other biomarkers, such as creatinine, are also enzyme substrates, the relevant enzymes do not produce easily detectable products, thus preventing their application to continuous, in vivo monitoring. And for many other targets, including many important drugs (see ref. [6] for a rare counter example), no enzymes are available. Given this, there remains a pressing need for strategies that are capable of in vivo molecular monitoring that are “agnostic” to the chemical or enzymatic reactivity of their targets. To this end, we, and others [7,8,9], are developing electrochemical aptamer-based (EAB) sensors: the first “platform” molecular monitoring technology that is both (i) independent of the chemical reactivity of its targets, and thus generalizable to new targets, and (ii) able to measure molecular concentrations in situ in the body (Figure 1). Building on these strengths, EAB sensors have already been shown to support seconds and even sub-second resolved measurement of more than a half dozen drugs and metabolites in the plasma (intravenous) [3,10,11,12,13], cerebrospinal fluid (brain tissue and ventricles [4]), and ISF (subcutaneous, intramuscular, intra-tumor [7,8,9]) of live rats, and have even used the resulting real-time concentration information to perform closed-loop feedback control drug concentrations in the plasma [3,14] and cerebrospinal fluid [4].

With the unique ability of EAB sensors to support real-time measurements of arbitrary molecules in the body notwithstanding, the intravenous placements we have largely employed to date are presumably too invasive for application outside of hospital settings. That is, to see a wider application, the technology likely must be adapted to performing measurements in the subcutaneous or intradermal ISF, which, by dramatically reducing invasiveness, would pave the way to real-time molecular measurements performed on patients going about their daily lives. As noted above, however, to achieve this clinical vision will require an improved understanding of how measurements performed in the ISF inform us on molecular concentrations in the plasma, as the latter are the basis of effectively all clinical decision making. To address this need, here we have used simultaneous intravenous and subcutaneous EAB sensor placements to determine the disposition kinetics of the antibiotic vancomycin from the plasma to the ISF of live rats with unprecedented, few-seconds resolution.

## 2. Results

### 2.1. Vancomycin EAB Sensors

EAB sensors comprise a target-recognizing aptamer that is modified with a methylene blue redox reporter and attached to an underlying electrode via thiol-on-gold self-assembled monolayer chemistry (Figure 1A). The aptamer is engineered such that it undergoes a conformational change upon target binding, altering the rate of electron transfer from the reporter and producing an easily measurable signal when the sensor is interrogated electrochemically, such as with square wave voltammetry (Figure 1B). In the work described in this paper, we adapted a previously described EAB sensor against the antibiotic vancomycin [3] to subcutaneous placements by fabricating them on a 200 μm diameter, 6 mm-long gold working electrode, which we bundled with a 200 μm by 1 cm (exposed) silver/silver chloride reference electrode in a 22 G catheter. To complete the three-electrode set up, we employed a 250 μm by 15 mm (exposed) platinum counter electrode placed in a separate 22 G catheter (Figure 1C). To access the ISF of the subcutaneous space, we used needles to insert two shielded 18 G “guide” catheters (placed within ~2 cm of one another) ventrally, just under the skin at a shallow angle, such that the luminal opening is at a depth of a few millimeters below the surface of the skin (Figure 1C). We did this by lifting the skin of the animal and inserting the needle at an angle that was parallel with the skin surface, as would be done for a subcutaneous injection. After insertion, we removed the needle and trimmed the top portion of the catheter, leaving the catheter tubing projecting into the subcutaneous space. We then passed the smaller gauge catheter containing the sensor and its reference electrode through one of the guides and the catheter containing the counter electrode through the other, such that the three electrodes project directly into the subcutaneous space. To ensure that our sensors are measuring drug concentrations in the ISF, rather than in the plasma (i.e., that insertion did not cause significant bleeding at the sensor), we monitored for obvious blood during insertion and then examined both the sensor and guide catheter for the presence of blood and/or blood clots after the termination of the experiment. Fortunately, we have never observed blood flow during insertion, and have observed blood or blood clots on the probe only on very few occasions (less often than once per 10 subcutaneous insertions). We excluded these from our study measurements performed using sensors on which the latter was observed to occur.

EAB sensors perform well when deployed in the subcutaneous ISF. As is likewise true for intravenous EAB placements, however, achieving this requires drift correction, which we performed by collecting measurements at square-wave frequency pairs that drift in concert but respond differentially to the presence of the target [12]. Taking the difference between the two, an approach termed kinetic differential measurements (KDM) corrects for in vivo sensor degradation (Figure 1D), leading to the expected return to baseline signal as the drug is eliminated from the body (Figure 1D). After this drift correction, the subcutaneous EAB sensors employed here achieve a temporal resolution of ~27 s and concentration resolution (root-mean-squared, pre-injection baseline noise) of, typically, ~1 μM.

### 2.2. Simultaneous Subcutaneous and Intravenous Measurements

**Figure 2:**
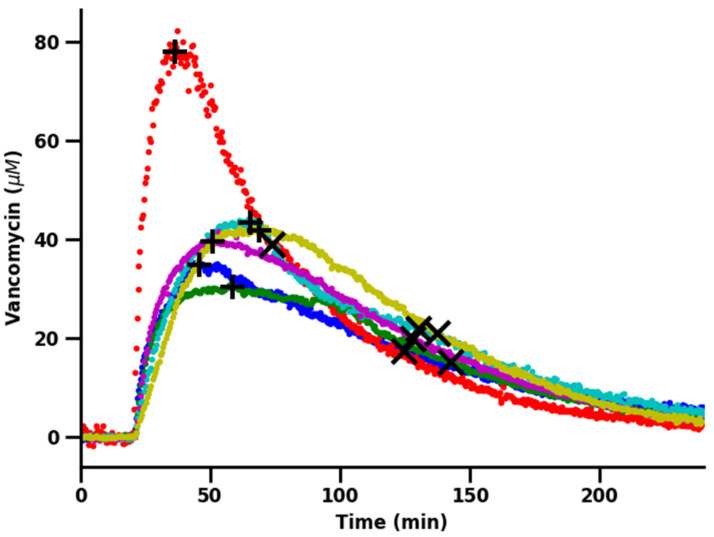
Vancomycin pharmacokinetics in the subcutaneous ISF vary significantly, even when animals are challenged with the same intravenous dose. To illustrate this, we here present the time-resolved drug concentrations measured in the subcutaneous ISF of 4 animals (2 of which have 2 subcutaneous sensor placements) after each was challenged with the same 30 mg/kg (intravenous delivery over 1 min) body-mass-adjusted dose of the drug. In each case, the sensors were placed just below the skin on the ventral portion of the animal (Figure 1C). $C_{max}$ and $C_{1/2}$ (after $C_{max}$) points are demoted by “+” and “x,” respectively. These values can be found in Table S1.

We performed simultaneous ISF and plasma measurements in four animals by placing one sensor in the right ascending jugular vein [3,10,12] and at least one sensor in the subcutaneous space (Figure 1C). Two animals received a second subcutaneous sensor placement to challenge site-to-site differences. Challenging the animals with 30 mg/kg vancomycin (infused over 1 min), the resulting 6 ISF concentration time courses exhibit biphasic pharmacokinetics, as the drug is first transported from the blood to the ISF before moving back from the ISF to the blood as the drug is eliminated via the kidneys (Figure 2). Specifically, upon intravenous challenge with vancomycin, drug concentrations in the subcutaneous ISF rise to a maximum concentration, $C_{max}$, of 30.3 to 78 µM (43.4 to 111.8 mg/L) over the course of 16.8 to 48.3 min (Table S1). This phase is then followed by a slower decrease in ISF drug concentrations with half-lives of approximately 37.4 to 84.2 min. We obtained these values in a model-free manner by performing a moving average over 10 measurements, using the maximum value of this average as $C_{max}$ and the point on the smoothed curve the concentration falls through as half of $C_{max}$.

**Figure 3:**
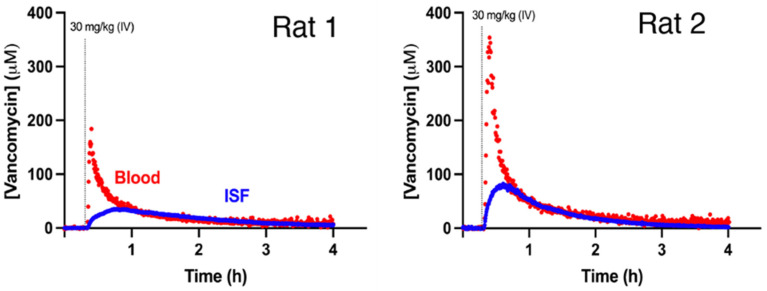
Simultaneous intravenous and subcutaneous vancomycin measurements confirm that inter-animal variability in the drug’s plasma pharmacokinetics that arises even when, as here, the dosing is adjusted for differences in body mass likely account for much of the inter-animal pharmacokinetic variability we observe in the ISF. To collect these data, we placed an intravenous sensor [15] in the right jugular vein and a subcutaneous sensor just below the skin on the ventral portion (Figure 1C) of each individual animal. The 30 mg/kg vancomycin challenge was delivered intravenously over a 1 min infusion using an indwelling jugular catheter.

**Figure 4:**
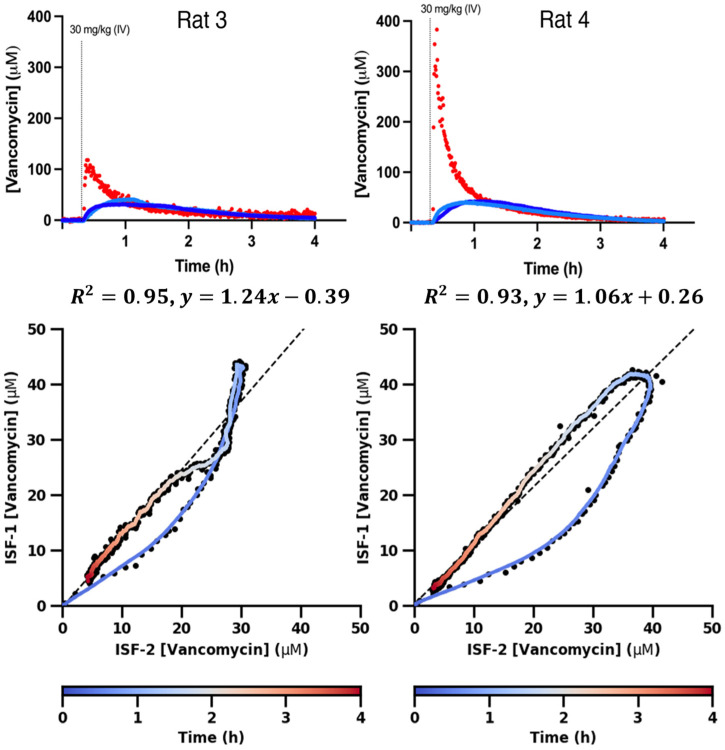
(**Top**) Although, given the small sample size, these data must be considered preliminary, vancomycin pharmacokinetics in the subcutaneous ISF appear to vary only slightly from placement to placement. To demonstrate this, here we placed pairs of subcutaneous sensors contralaterally in the ventral portion of individual animals, with each working electrode placed roughly equidistant from the platinum counter electrode (Figure 1C). The animals were dosed to 30 mg/kg over a 1 min infusion. (**Bottom**) While not identical (presumably due to small site-specific differences in the rate of transport from the blood), the concentration time courses measured at the two sites are quite similar. To show this, here we have plotted the ISF concentrations against each other (black dots) and fitted a linear relation between the concentrations (smoothed using a 10-point rolling average) measured at the two sites (dashed line). For the same analysis with unsmoothed data, see Figure S1, and for the fitted linear regression parameters, see Table S2.

The ISF vancomycin pharmacokinetics we observed differ from rat to rat, despite our having employed body-mass adjusted dosing in similarly aged animals (Figure 2). Some of this variation arose due to animal-to-animal variation in plasma pharmacokinetics [3]. Specifically, when we compared the plasma pharmacokinetic profiles collected (at the same time as the above ISF measurements) in the right ascending jugular vein [3,10,12], we observe nearly two-fold variation in plasma $C_{max}$ (Figure 3 and Figure 4). This said, some of the variation may also be due to site-to-site variations in ISF pharmacokinetics, a question we address in detail below.

If ISF pharmacokinetics were a strong function of where in the subcutaneous space the measurements were performed, achieving our goal of minimally invasive therapeutic drug monitoring would prove difficult. Fortunately, however, the variability between different subcutaneous placements in the same animal is a relatively small contributor to the variable inter-animal subcutaneous pharmacokinetics observed above (Figure 4, top). To see this, we performed measurements simultaneously in the plasma and at two subcutaneous sites 1 to 2 cm apart on the ventral surface of two animals. Doing so, we found that, while not identical (presumably due to site-specific differences in the rate of drug transport), and limited in number, the concentrations seen at pairs of subcutaneous sites within individual animals are quite similar, and remain fairly well correlated across the entire drug elimination time course (Figure 4, bottom).

### 2.3. Correlation Between Plasma and Subcutaneous ISF Concentrations

**Figure 5:**
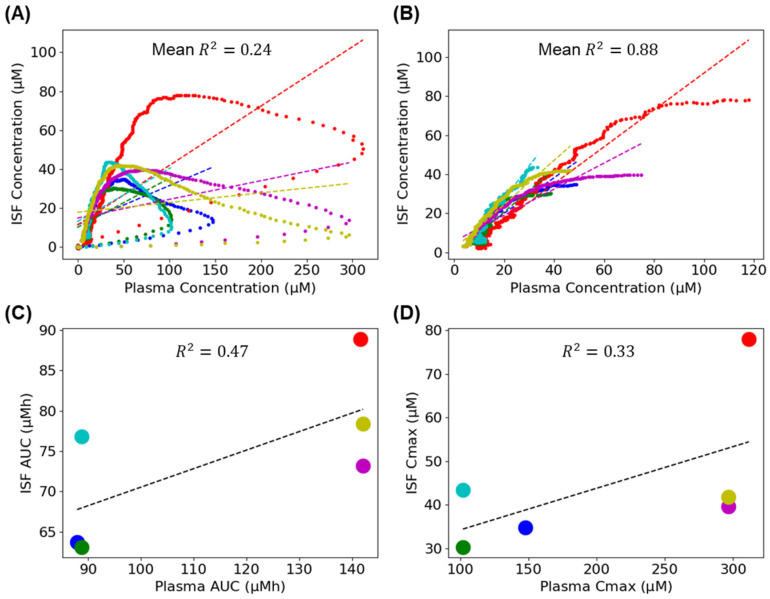
A statistical look at the relation between the plasma and the ISF measurements. Here, we study the smoothed measurements (using a moving average smoothing with window of 10 points) without any modeling assumptions. (**A**) At the high temporal and concentration resolution EAB sensors achieve, the correlation between plasma and ISF drug concentrations is only modestly correlated (with an average $R^{2}$ value of 0.24; see Figure S2 for details). Given that drug concentrations peak much higher and much more rapidly in the plasma peaks in the ISF, this should be expected. (**B**) To counteract this initial divergence, we can remove the measurements before the concentration peaks in ISF (on average after 55.4 min). Under this regime, drug elimination phases in the plasma and in the ISF are much more strongly correlated (average $R^{2}$ value of 0.88; see Figure S2 for details of the individual curves). (**C**) Measures of drug exposure (area under the curve; AUC) and (**D**) $C_{max}$ are, likewise, only modestly correlated between the two bodily fluids. This said, our experiments captured only a narrow range of pharmacokinetics (all animals were challenged with the same body-mass-adjusted dose), which would weaken any such correlations. For the same analysis with unsmoothed data, see Figure S3, and for all the linear fit coefficients, see Table S2.

Previous studies using microdialysis and microneedles to collect subcutaneous ISF and blood draws to sample plasma identified modest, but statistically significant correlations between vancomycin concentrations in these two bodily fluids [16,17]. However, due to the unprecedented time resolution with which we captured the pharmacokinetics in the two compartments, these correlations are rather poor in our study (Figure 5A). That is, the prior work collected samples over 10 min, with the resulting poor time resolution causing them to significantly underestimate $C_{max}$ in the plasma. When we accurately captured this peak, which happens much earlier in plasma than in the ISF, the drug concentrations in the two compartments were no longer well correlated. To counteract the divergence between the plasma and ISF drug concentrations during and immediately after infusion, we could remove measurements collected before the concentration peaks in the ISF (which occurs, on average, 34.1 min after the infusion start). Under this approach, which looks only at the drug eliminations and not the rising phases, the concentrations in the plasma and ISF were much more strongly correlated (mean $R^{2}$ value of 0.88; Figure 5B). Given that trough measurements remain the most commonly used approach to vancomycin TDM [18], this suggests that subcutaneous EAB sensors may provide a viable route to achieving the minimally invasive, real-time monitoring of this important and difficult-to-dose antibiotic.

Drug exposure (“area under the curve” = AUC) and $C_{max}$ are also positively correlated (R^2^ = 0.47 and 0.33, respectively) between the two measurement sites (Figure 5C,D), albeit more weakly than has been reported in prior studies of subcutaneous and plasma tobramycin [19] and doxorubicin [20] in rats. This discrepancy presumably arose due to the single body-mass-adjusted drug dose (30 mg/kg in all rats) we employed here, which leads to far narrower AUC and $C_{max}$ ranges than those seen in the prior literature, which used variable doses, and limited our ability to generalize across a wide range of doses.

### 2.4. Compartmental Modeling

**Figure 6:**
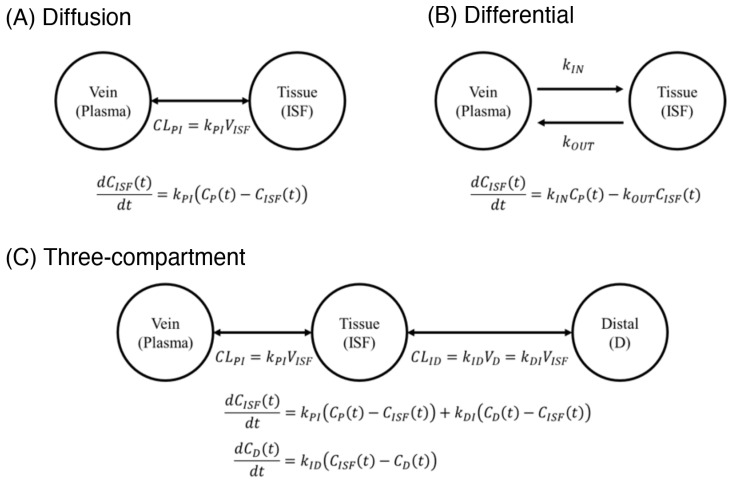
The availability of simultaneous, high-time-resolution drug measurements in the blood and ISF provides a unique opportunity to explore the pharmacokinetics of drug transport into the tissues. Here, we have done so using three models. (**A**) The first, a two-compartment “diffusion model,” assumes that the vein (filled with plasma) and the solid tissue (filled with ISF) are the only two relevant compartments, and that transport between them is driven by simple, passive diffusion along concentration gradients. If, as is true here, the time-resolved concentrations in the plasma and ISF are both known, this model is defined by a single fitted parameter, $k_{PI}$: the rate constant that describes transport between the two compartments. (**B**) The next more complex model, the “differential transport model”, assumes an asymmetry in the forward and reverse transport kinetics between the two compartments, thus employing two fitted rate constants. (**C**) Finally, the most complex model we investigated, a “three-compartment diffusion model”, employs a third, “distal” compartment that is unobserved and that communicates with the plasma only via the ISF. This model also assumes passive, diffusive transport between the vein and solid tissue, and the tissue and this distal compartment.

The unprecedentedly high time resolution of EAB-sensor-derived, simultaneous plasma and ISF drug concentrations prompted us to next explore compartmental models of vancomycin transport between the two measurement compartments. To perform this modeling, we first fitted the plasma time courses to a two-compartment model (Equation (S1) and Table S4) and then the resulting plasma pharmacokinetic model to fit the ISF pharmacokinetic profiles that we measured. Specifically, we tested the ability of three increasingly complex pharmacokinetic models to describe our ISF observations. In the first, we assume that the plasma-filled veins and the ISF-filled “solid tissues” are the only significant compartments, and that the drug is transported between these via passive diffusion (Figure 6A). If, as is true here, for known time-resolved concentrations in the plasma, $C_{P}$, and ISF, $C_{ISF}$, as the “diffusion model,” is defined by a single fitted parameter, $k_{PI}$, that describes the impact of the material transport between the compartments onto the concentration in ISF (Equation (1)).

(1) $$\frac{{dC}_{ISF}\left(t\right)}{dt}=k_{PI}\left(C_{P}\left(t\right)-C_{ISF}\left(t\right)\right)$$

At the next level of complexity is a “differential transport model.” In this case, hypothetical physiological effects create a nonequivalence between the rate constant describing transport from the vein to the tissue, $k_{IN}$, and the constant describing transport from the tissue to the vein, $k_{OUT}$, thus yielding two fitted parameters (Figure 6B).

(2) $$\frac{dC_{ISF}\left(t\right)}{dt}=k_{IN}C_{P}\left(t\right)-k_{OUT}C_{ISF}\left(t\right)$$

Finally, the most complex model we employed, a “three-compartment diffusion model,” adds a third, “distal” compartment to the two-compartment passive diffusion model. This third compartment, the concentration in which we denote by $C_{D}$, is in direct, diffusive communication with the tissue, but only has indirect access to the vein via the tissue (Figure 6C). This model contains the fitted parameters $k_{PI}$ (transport from plasma to the ISF), $k_{DI}$ (transport from the distal compartment to the ISF), and $k_{ID}$ (transport from the ISF to the distal compartment).

(3) $$\frac{dC_{ISF}\left(t\right)}{dt}=k_{PI}\left(C_{P}\left(t\right)-C_{ISF}\left(t\right)\right)+k_{DI}\left(C_{D}\left(t\right)-C_{ISF}\left(t\right)\right)$$

(4) $$\frac{dC_{D}\left(t\right)}{dt}=k_{ID}\left(C_{ISF}\left(t\right)-C_{D}\left(t\right)\right)$$

Note that, despite the fact that transport between the ISF and the distal compartment is diffusive in this model, $k_{ID}$ and $k_{DI}$ are not equal due to the differing volumes of the ISF and distal compartments; this model thus contains three fitted parameters. In fact, the symmetric matter transfer implied by diffusion dynamics is usually reflected by clearance rates [21]. This symmetry is broken, however, when we convert equations into concentrations instead of total amount of drug.

**Figure 7:**
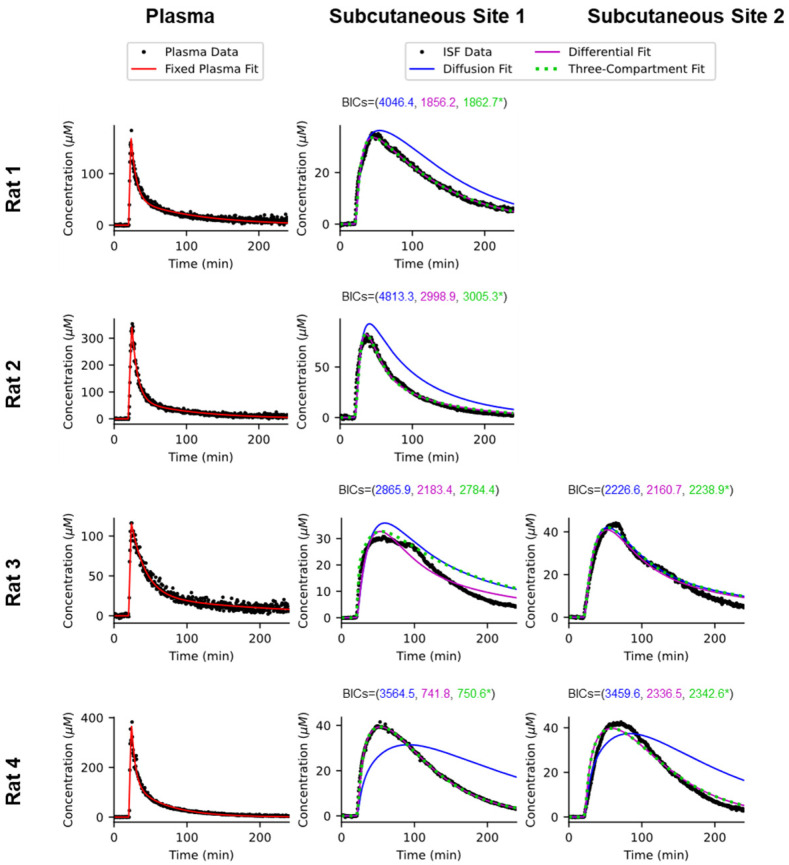
Here, we have modeled drug transport into and from the ISF by first fitting the plasma data (black dots, lefthand column) to a two-compartment model (red curves; Table 1). Using these as the true plasma concentration, we then fit our observed ISF measurements (black dots, middle and righthand columns) to three models. The first (blue curves) is a simple, passive diffusion model in which a single rate constant describes transport into and out of the ISF. The second (purple dashed lines) assumes that, due to differences in the solution conditions found in blood and plasma, the rate constants for transport into and out of the ISF are distinct. The third assumes that the ISF is an intermediate compartment that communicates with a third, distal compartment that is not observed. The Bayesian Information Criterion (BIC), a model selection tool that penalizes for model complexity [22], indicates that the differential transport model is the most appropriate description of our data for all six data sets. Indeed, for most of our data sets, the three-compartment compartment model, which contains an additional fitted parameter over the differential transport model, collapses onto the differential transport model (this is indicated by asterisks on three-compartment BIC values).

Although the simple, two-compartment diffusion model accurately tracks the seconds-resolved pharmacokinetics of vancomycin in the ISF, it nevertheless systematically fails to capture the details of these very high-time-resolution measurements (Figure 7, blue curves). For example, $C_{max}$ in the ISF is reached before the point at which the plasma and ISF drug concentrations hit parity in five of our six paired plasma/ISF datasets (Figure 3 and Figure 4), an observation that cannot be reconciled with this simple model. Specifically, for this model, the concentration seen in the ISF will rise until the point at which the ISF and plasma concentrations are equal, after which the ISF concentration will fall as the direction of the concentration gradient switches.

In contrast to the two-compartment passive diffusion model, and despite containing only two fitted parameters, the two-compartment differential transport model fits most of our paired plasma/ISF data sets with sometimes startling precision (Figure 7, magenta curves). In rat 1, for example, the differential transport model fits the measured ISF concentrations with a root mean squared error (RMSE) of just 0.97 μM, a value close to the root-mean-squared baseline noise we observe prior to the drug challenge. The model achieved similar (RMSE = 0.53 μM) precision for one of the two simultaneous ISF datasets we collected in rat 4. For our other four data sets (rat 2, two in rat 3, one in rat 4), the differential transport model deviates more significantly from our observations. But even for these more poorly fitting data sets, the RMSE of the differential transport model remains quite good (between 2.5 and 3.1 μM), and for every measurement dataset, the fit of the two-compartment differential transport model is notably better than that of the two-compartment diffusion model, see Table S5 for all the metrics.

As our next exploration, we fit our data sets to the above-described three-compartment models, in which there is an unobserved compartment distal to the (observed) interstitial fluid (Figure 6C). Doing so, we find that the precision with which the two-compartment differential transport model fits our data sets is so great that, despite its containing one more fitted parameter than the differential transport model, the three-compartment model generally does not fit our time-dense subcutaneous measurements any better than the simpler, differential transport model. For example, in five of our six pair data sets, the best fit of the three-compartment model assigns one of the parameters a value that is infinitesimally close to 0 (Table 1), rendering the best fit of this model indistinguishable from the differential transport model (i.e., in Figure 7 the green three-compartment model is perfectly superimposed over the magenta differential transport model for five of our six paired plasma/ISF measurements). Consistent with this, the Bayesian Information Criterion (BIC), a measure of model appropriateness that weighs goodness of fit against model complexity, identifies the differential transport model as the most appropriate of the three models investigated for all six of our paired data sets (Figure 7). The results of our modeling indicate that, in addition to variable plasma pharmacokinetics, animal-to-animal changes in tissue perfusion also contribute significantly to the variable ISF pharmacokinetics we observe across animals. Specifically, while the half-dozen paired plasma/ISF data sets we have collected here reflects only a preliminary survey, they already suggest that the inter-animal variability in the rate constants for transport into ($k_{IN}$) and out of ($k_{OUT}$) the ISF is significant; these vary by up to a factor of three between our test-bed animals (Table 1, and for approximated standard errors, see Table S6). In contrast, within individual animals, the variation in these parameters between different subcutaneous sites is far smaller, thus accounting for the between-site correlations we observe in the ISF (Figure 4, bottom).

## 3. Discussion

Using EAB sensors, here we have monitored the pharmacokinetics of the drug vancomycin in the subcutaneous ISF with a few-second resolution. Doing so, we find that the resulting drug concentration—time profiles vary significantly between animals given the same body-mass-adjusted dose (Figure 2). This variation arises due to both animal-to-animal differences in plasma pharmacokinetics (Figure 3) and in the rate of transport from the blood to the ISF (Table 1). In contrast, inter-site differences within individual animals contribute relatively little to the variable pharmacokinetics (Figure 4), a conclusion that we also observed in healthy humans [19]. Exploring noncompartmental models of these simultaneous, highly time-resolved measurements uncovers strong correlations (average *R^2^* = 0.88) between drug concentrations in the two compartments once the most rapid pharmacokinetic phases have approached equilibrium (i.e., after $C_{max}$ is reached in the ISF). More detailed, compartmental models of transport between the plasma and the ISF indicate that, while the relationship between the drug time courses in the two compartments is slightly, but systematically, mis-described by a passive diffusion, two-compartment model, for most of our data sets it is fitted exceptionally well by a two-compartment differential transport model. This is so much so that, despite including an additional fitted parameter, a three-compartment model encompassing a compartment that is more distal to the ISF does not fit our data any more accurately than the two-compartment differential transport model.

While with only four animals, our study can only be considered a preliminary survey, the fact that the differential-transport two-compartment model fits all of our data sets quite well and fits several of them with an exceptionally high level of precision mirrors suggests that it provides an impressive accurate representation of vancomycin’s transport to and from the solid tissues, at least in the small number of animals we have characterized here. This result is also consistent with recent findings regarding vancomycin in healthy human subjects [19]. Despite the inter-species consistency, these findings somewhat challenge the prevailing notion that the primary route of communication between the plasma and ISF is via the relatively large gaps that occur between the cells lining the finest capillaries [23]. The lack of any membrane barrier between the two compartments argues that the differing in and out rate constants we observe are not due to transporter-mediated active transport. Likewise, as our sensors presumably only respond to free vancomycin, differential protein binding cannot explain this difference in the predicted equilibrium concentrations (if the protein-bound drug is invisible to the sensor, the difference in the two concentrations is the difference in the free drug concentration, not the total drug concentration). Given that vancomycin has an ionizable group with a pK_a_ near pH 7, however, this transport discrepancy may arise due to the small, metabolism-generated pH gradient that occurs between the tissues and the blood [24,25,26], which could produce the small, asymmetric distributions we observed in the equilibrium distribution between the two bodily fluids. Conversely, this discrepancy could be due to small errors in the calibration (sensor-to-sensor reproducibility in these handmade devices is of an order of 10% [27]), or to calibration errors arising from differences in core versus peripheral body temperatures. 

Comparing the present results with earlier data collected in clinical trials [19] reveals substantially faster ISF transport kinetics in rats (1.46±0.66 $h^{-1}$ and 2.01±0.91 $h^{-1}$ for $k_{IN}$ and $k_{OUT}$ respectively) than in humans (0.21±0.05 $h^{-1}$ and 0.18±0.09 $h^{-1}$, respectively). This could be due to the generally higher metabolic rates of rats, which also extends to their more rapid drug elimination. For example, while we see the almost total elimination of plasma vancomycin in rats in 3 to 4 h, this required ~12 h in healthy humans.

The results of our modeling aside, the model-free observation that inter-site variation in the subcutaneous pharmacokinetics of vancomycin are relatively small is both expected and important. It is expected because all sites in the subcutaneous space presumably have similar metabolic needs; thus, we expect that, for any given animal, the rate of transport of small molecules to and from the blood to all sites in the subcutaneous space will be similar. Consistent with this, the site-to-site variation in subcutaneous ISF pharmacokeintics is also small for the drug doxorubicin [20]. This observation is important because significant site-to-site variability would render it difficult to employ subcutaneous measurements to accurately estimate plasma pharmacokinetics. That is, the small size of this variation suggests that it may prove possible to use minimally invasive, highly time-resolved, real-time drug concentration measurements performed in the ISF to significantly advance therapeutic drug monitoring, which has historically relied on, at most, one or two time points per drug administration and typically only returns actionable information to the clinician hours or days after sample collection [28,29].

Regarding the use of subcutaneous drug concentration measurements, which can be accessed much less invasively than plasma [30], to support therapeutic drug monitoring, we note that the strength of the correlation between molecular concentrations in the plasma and ISF depends on the ratio of two timescales: the timescale over which clinically meaningful changes in plasma concentration occur and the timescale for diffusion from the plasma into the ISF. For glucose, which has a low enough molecular weight that it diffuses into tissues rapidly and undergoes relatively small (typically less than 2-fold), slow plasma fluctuations (digestion, tissue uptake, gluconeogenesis, and glycogenolysis occur on tens), the ratio of these timescales is favorable, and thus continuous glucose monitors more or less simply assume that the two concentrations follow one another with a short lag time [27]. For vancomycin, in contrast, the situation is far less optimal. Specifically, as an intravenously administered drug, plasma vancomycin concentrations fluctuate much more rapidly and significantly (e.g., upon delivery of the first dose, the concentration rapidly rises from 0 to $C_{max}$). Likewise, due to its 10-fold higher molecular weight, vancomycin presumably diffuses into tissues more slowly than glucose. Despite this, after $C_{max}$ is reached in the ISF, the average correlation between plasma and ISF vancomycin concentrations is *R*^2^ = 0.88. Given that trough measurements remain the most commonly used approach to vancomycin TDM [18], this suggests that subcutaneous EAB sensors may provide a viable route to achieving the minimally invasive, real-time monitoring of this important and difficult-to-dose antibiotic.

## 4. Materials and Methods

### 4.1. Chemical Reagents and Materials

We employed that following chemical reagents: phosphate-buffered saline (PBS) was diluted from a 20× stock (Santa Cruz Biotechnologies, Santa Cruz, CA, USA), sulfuric acid, sodium hydroxide, 6-mercapto-1-hexanol, and tris(2-carboxyethyl)phosphine (Sigma Aldrich, Burlington, MA, USA), Vancomycin HCl (VWR, Visalia, CA, USA) and Methylene blue and HO-C6 S-S-C6-modified DNA sequences (sequence from ref. [3]; Integrated DNA Technologies, Coralville, IA, USA).

### 4.2. Sensor Fabrication

For intravenous sensors, gold (0.2 µm diameter × 10 cm length, 99.9% purity, A-M Systems), platinum (0.125 µm diameter × 10 cm length, 99.95% purity, A-M Systems), and silver (0.125 µm diameter × 10 cm length, 99.99% purity, A-M Systems) wires were insulated with polytetrafluoroethylene heat-shrink (PTFE, HS Sub-Lite-Wall; Zeus Inc., Orangeburg, SC, USA). The wires were bundled with physical gaps to prevent shorting and the insulation was trimmed to expose 3 mm (gold), 5 mm (platinum), and 1 cm (silver). The silver wire was converted to a reference electrode by submerging it in 7.5% sodium hypochlorite (commercial bleach, Clorox, Oakland, CA, USA) overnight to form a stable silver chloride film, followed by rinsing it in deionized (DI) water to remove the residual bleach.

For subcutaneous sensors, gold (0.2 µm diameter × 10 cm length) and silver wire (0.2 µm diameter × 10 cm length, 99.99% purity, A-M Systems, Sequim, WA, USA) were insulated with polytetrafluoroethylene heat-shrink (PTFE HS Sub-Lite-Wall). The insulation was trimmed to expose 5 mm (gold) and 1 mm (silver). The silver wire was converted to a reference electrode by submerging it in 7.5% sodium hypochlorite overnight, followed by a DI water wash. A separate counter electrode was fabricated by heat-shrinking PTFE platinum wire (0.25 µm diameter × 10 cm length) and feeding it through a 20-gauge catheter.

Both intravenous and subcutaneous electrodes were functionalized into sensors as follows. The disulfide bond in the methylene-blue-modified DNA was reduced with 14 mL of 10 mM tris(2-carboxyethyl)phosphine and 2 mL of 100 mM DNA for 1 h in the dark. After assembling and overnight bleaching, the sensors were rinsed with DI water and cleaned by:

1. Cycling the potential 1000 times between −1.0 and −2 V versus Ag|AgCl in a 0.5 M NaOH solution (1 V/s) to remove residual organic or thiol contaminants.
2. Pulsing between 0 and 2 V by applying 32,000 20 ms pulses in 0.5 M H_2_SO_4_ to increase the electrode surface area.
3. Roughening the electrode surface by cycling the potential between 1.5 and −0.35 V at 1 V/s four times in H_2_SO_4_.

The gold electrodes were then rinsed in DI water, fed through 20-(intravenous) and 22-(subcutaneous) gauge catheters, and immersed in 500 nM reduced DNA dissolved in PBS for 1 h. The sensors were then transferred to a 10 mM solution of 6-mercapto-1-hexanol in PBS overnight at room temperature to complete the formation of their self-assembled monolayers. The intravenous and subcutaneous sensors, along with the external counter of the latter, were fed through the lumen of 22- and 20-gauge catheters, respectively (Becton Dickinson & Company, Franklin Lakes, NJ, USA). Before their use in vivo, the catheters were filled with 1× PBS.

### 4.3. Sensor Interrogation

The sensors were interrogated electrochemically by using square-wave voltammetry on a CH1040C multipotentiostat (CH Instruments Inc, Bee Cave, TX, USA). Calibration parameters were determined by performing a 28-point titration in 37 °C bovine blood at “signal-on” and “signal-off” frequencies of 100 and 30 Hz, respectively. Drift correction was then performed using kinetic differential measurements (KDM), obtained by taking the difference in normalized peak currents collected at our signal-on and signal-off frequencies:

(5) $$KDM=\frac{\left({Signal}_{on}-\;{Signal}_{off}\right)}{\frac{1}{2}({Signal}_{on}+\;{Signal}_{off})}$$

To fit the resulting KDM signals to drug concentrations, we fit in vitro titration data to the following equation:

$$\mathrm{KDM}\;=\;{\mathrm{KDM}}_{\min}\;+\;\frac{({KDM}_{max}-{KDM}_{min})*{\left[Target\right]}^{n_{H}}}{{\left[Target\right]}^{n_{H}}+{K_{d}}^{n_{H}}}\;$$

where ${KDM}_{max}$ is the maximum signal gain observed at saturating concentrations, [Target] is the drug concentration, *n_H_* is the Hill Coefficient, and K_d_ is the binding half-point of the aptamer. We employed blood as the calibration matrix for both intravenous and subcutaneous sensors, using the argument that the walls of the finest capillaries are quite leaky (molecules under 10 kDa pass through quite readily) [23], and thus plasma is a good proxy for the calibration of sensors to be deployed in the ISF. To be precise, in this work, we used $n_{H}=1$, $KDM_{min}=0$, $KDM_{max}=0.49$, and $K_{d}=64.94\;\mu M$. In support of this argument, we have recently shown that, when placed in the subcutaneous ISF of live rats, doxorubicin-detecting EAB sensors calibrated in this same manner achieve identical accuracy to intravenous sensors calibrated in blood [20].

### 4.4. Correlation Analysis and Compartmental Modeling

We performed correlation analysis using the standard linear regression function under SciPy [31] (stats.linregress). This standard regression function readily provides the estimated standard errors and the associated Pearson correlation coefficient of which we take the square to report all the coefficient of determination values ($R^{2}$) reported throughout this work.

We use compartmental models to explain our data in a stepped manner. We first fit the plasma data to a two compartmental model (Equation (S1)). Then, we fit the diffusion (1), differential (2), and the distal (3, 4) drug transport models. These models are all structurally identifiable (see the Structural Model Identifiability Section in Supplementary Materials). We then fit the models to the data using minimization of the sum of squares of residuals.

(6) $$\underset{\theta \in \Theta }{\min}\sum_{i=1}^{N}{\left(y\left[i\right]-f\left(t\left[i\right];\theta \right)\right)}^{2}$$

where t[i] and y[i] are the $i^{th}$ time and the unsmoothed concentration measurement pair, θ is the parameter values, Θ is the feasibility set for θ values that includes positivity constraints, Nyquist frequency-based maximum-parameter values for the plasma fits and a positive discriminant constraint for the second order systems to ensure distinct real roots, and $f\left(t\left[i\right];\theta \right)$ is the function denoting the estimated concentration value at time $t\left[i\right]$ and given θ values. We use the same structure for all our optimizations, with differing calculation functions, f. This structure also allows us to calculate the approximate parameter covariance matrix.

Under the assumption that the estimated parameter values $\hat{\theta }$ obey a Gaussian a posteriori distribution, the covariance matrix can be approximated by the inverse of the Hessian of a maximum likelihood estimation [32] for a log-likelihood maximization problem with zero-mean Gaussian noise assumption. Here, we do not use the full maximum likelihood estimation formulation and instead minimize the sum of squared errors (6). However, under the assumption of independent Gaussian residuals, minimizing the sum of squared residuals for a given dataset yields the same parameter estimates as maximum-likelihood estimation with a dataset-specific noise variance. Because we fit one dataset at a time, that variance does not need to be modeled jointly across datasets. In other words, the noise variance estimation does not have any impact on the rest of the parameters. Furthermore, we can use the Hessian of the minimization (6) by augmenting it with the appropriate observation noise variance.

(7) $$Cov\left(\hat{\theta }\right)\approx 2{\hat{\sigma }\left(\hat{\theta }\right)}^{2}{H\left(\hat{\theta }\right)}^{-1}$$

where $\hat{\sigma }\left(\hat{\theta }\right)$ is the residual standard deviation of the residuals, and $H\left(\hat{\theta }\right)$ is the Hessian (second derivative) of the sum of residuals cost (6) calculated for the estimated parameter set $\hat{\theta }$, and the scaling factor of two comes in due to the ignored factor of 2 in the real maximum likelihood estimation cost. Also note that when we utilize a parameter reformulation, such as in (S9) and (S12), we also apply the appropriate Jacobians to account for change in the parameters. The results of these approximated errors can be found in Tables S4 and S6.

While the structural identifiability of our models is established theoretically (see Structural Model Identifiability Section in Supplementary Materials), this is different from practical identifiability. Indeed, we observed that the distal model only fit meaningfully for Rat-3-1. For all other datasets, the fitted parameter values were effectively zero, indicating a lack of practical identifiability in those cases. This observation is further supported by the poor reciprocal Hessian condition numbers (see Table S5).

We used a stepped approach to fitting our datasets. While this simplifies the optimization by avoiding the simultaneous noise variance estimation, it means that the uncertainty in the plasma parameter estimation is not propagated to the ISF estimations. To test that the uncertainty in plasma estimation does not affect our final results, we ran an empirical bootstrap method by sampling the plasma parameter space, using the estimated parameter values as the mean and the estimated covariance matrix (7) as the covariance of the sampling distribution. The resulting sampled parameters are used to create new fixed plasma traces, which are then used in the original ISF estimation routine. This is done 500 times per rat and the results show that our diffusion and differential model parameter estimates remain nearly identical (Table S7). This is not surprising, considering how well the original fits were conditioned (Table S5) and the relatively small estimated standard errors around the plasma estimates (Table S4). On the other hand, the distal model was only run for Rat-3-1, since all the rest had near-zero estimates to begin with (Table 1).

The numerical optimizations were performed using the algorithmic differentiation tool of CasADi [33] with the interior point method solver IPOPT [34].

### 4.5. In Vivo Measurements

Experiments were conducted using adult male Sprague-Dawley rats (4–5 months old, 300–500 g; Charles River Laboratories, Hollister, CA, USA). The rats were pair-housed in a temperature and humidity-controlled vivarium on a 12 h light–dark cycle, with ad libitum access to food and water. All procedures followed NIH guidelines and were approved by the Institutional Animal Care and Use Committee (IACUC) of the University of California Santa Barbara.

The rats were anesthetized with 4% isoflurane gas in a Plexiglas chamber, and anesthesia was maintained with 2–3% isoflurane gas/oxygen via a nose cone. Heart rate and SpO_2_ were monitored using a pulse oximeter (Nonin Medical, Plymouth, MN, USA). The skin above the jugular vein was disinfected, and a small incision was made to isolate both veins. A silastic catheter was inserted into the left jugular vein for infusions, and the EAB sensor was inserted into the right jugular vein for drug monitoring, stabilized with sterile 6-0 silk sutures. Following insertion, 30 units of heparin were infused through the indwelling line. The subcutaneous sensor and external counter electrode were inserted using an 18-gauge catheter just below the skin on the posterior ventral side between the legs.

A 0.01 M stock of vancomycin HCl was prepared using sterile saline and administered using a motorized syringe pump (KDS 200, KD Scientific, Holliston, MA, USA). Measurements were taken using square-wave voltammetry at square wave frequencies of 30 and 100 Hz over a voltage window of approximately −0.1 to −0.45 V (relative to the Ag|AgCl reference). Drift correction was performed using KDM. A 20 min baseline was established before infusing vancomycin. Once a stable sensor baseline was achieved, vancomycin (30 mg/kg) was injected over 1 min. The target concentration was quantified by fitting KDM values to the above-described Hill–Langmuir equation, with parameters obtained from in vitro calibrations in bovine blood at 37 °C.

## Figures and Tables

**Table 1:** Fitted pharmacokinetic parameters (in min^−1^).

	Parameter	Rat 1	Rat 2	Rat 3-1	Rat 3-2	Rat 4-1	Rat 4-2
ISF Diffusive	$k_{PI}$	0.024	0.036	0.023	0.031	0.006	0.008
ISF Differential transport	$k_{IN}$	0.026	0.040	0.025	0.031	0.012	0.011
	$k_{OUT}$	0.034	0.062	0.032	0.033	0.023	0.018
ISF Three-compartment	$k_{PI}$	0.026	0.040	0.184	0.031	0.012	0.011
	$k_{DI}$	0.008	0.022	0.960	3.2 × 10^−5^	0.010	0.007
	$k_{ID}$	5.1 × 10^−11^	4.9 × 10^−12^	0.128	2.94	6.7 × 10^−5^	1.2 × 10^−11^

## Data Availability

Correspondence and requests for materials should be addressed to kwp@ucsb.edu or kippin@ucsb.edu.

## Supplementary Materials

The following supporting information can be downloaded at: https://www.mdpi.com/article/10.3390/s26072233/s1, Figure S1: Shown is the ISF-to-ISF correlation analysis presented in Figure 4, without data smoothing. The fitted linear regression parameters are presented in Table S2; Figure S2: If we include the entire subcutaneous time course (i.e., including the extremely rapidly changing plasma drug concentrations seen before C_max_; black dots), the correlation between plasma and ISF drug concentrations are relatively poor (the black dashed lines). If, instead, we only consider the period after the most rapid pharmacokinetic phases have largely equilibrated (i.e., after C_max_ has been achieved in the ISF), the correlation between plasma and ISF drug concentrations becomes quite strong (colored dashed lines). The individual plots shown here use the same color employed in Figure 2 and Figure 5. The details of the linear fits can be found in Table S3; Figure S3: There is not much of a difference between using smoothed (top row) and “raw” data (bottom row) for the correlation analysis presented in Figure 5. The biggest difference is observed for $C_{max}$ values, which is expected, as $C_{max}$ is the most sensitive to smoothing operations. The detailed linear fit parameters can be found in Table S3; Table S1: Pharmacokinetic values; Table S2: The slope and intercept values using smoothed versus raw concentration data, visualized in Figure S1; Table S3: The slope and intercept values using smoothed vs raw concentration data, visualized in Figures S2 and S3; Table S4: Fitted plasma pharmacokinetic parameters; Table S5: ISF Modeling quality results for the parameters presented in Table 1; Table S6: The approximated standard errors for ISF parameter estimations. Fits with near-boundary estimates are ignored; Table S7: Bootstrap results. Fits with near-boundary estimates are ignored.
