# Supplementary material for: Seconds-Resolved Measurements of Vancomycin Transport from the Plasma to the Interstitial Fluid Highlight a Path Towards Real-Time Therapeutic Drug Monitoring
Source: Sensors (Basel). 2026 Apr 4;26(7):2233. doi: 10.3390/s26072233 (PMC13075304; doi:10.3390/s26072233)
Supplement: Supplementary file 1 [file sensors-26-02233-s001.zip › sensors-4211682-supplementary.pdf]

sensors

MDPI

Table S1. Pharmacokinetic values.

Subject	t_{max} (min)	C_{max} (µM)	$t_{1/2}$ after t_{max} (min)	$C_{1/2}$ (µM)
Rat-1	25.7	34.8	78.8	17.4
Rat-2	16.8	78.0	37.4	39.0
Rat-3-1	38.5	30.3	84.2	15.2
Rat-3-2	45.2	43.5	65.0	21.8
Rat-4-1	30.3	39.7	77.6	19.9
Rat-4-2	48.3	41.8	68.8	20.9

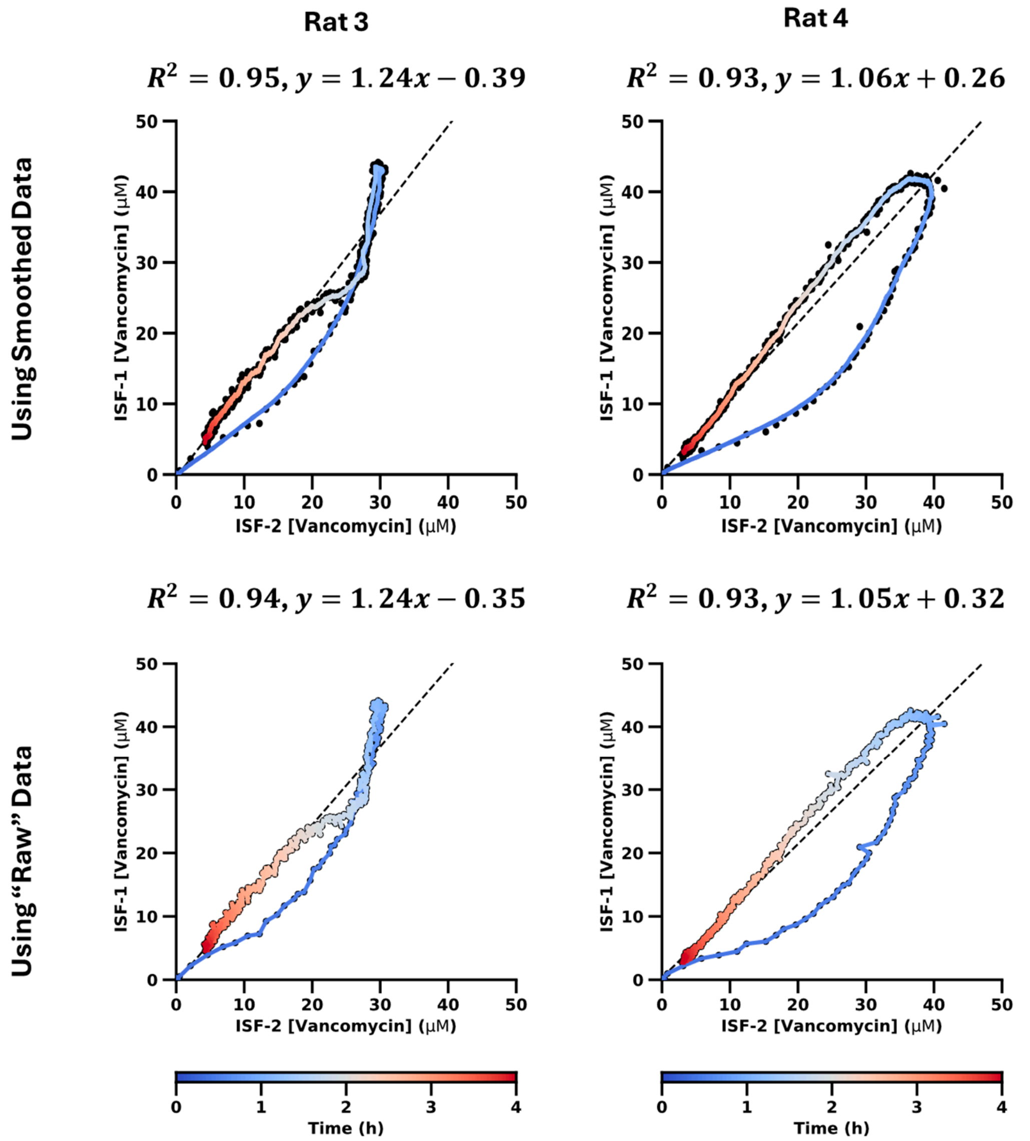

Figure S1. Shown is the ISF-to-ISF correlation analysis presented in **Figure 4**, without data smoothing. The fitted linear regression parameters are presented in **Table S2**.

Table S2. The slope and intercept values using smoothed versus raw concentration data, visualized in **Figure S1**.

	Smoothed Data					Raw Data				
	Slope	Stand. error	Intercept	Stand. error	R^2	Slope	Stand. error	Intercept	Stand. error	R^2
Rat-3	1.24	0.014	-0.39	0.26	0.95	1.24	0.014	-0.35	0.27	0.94
Rat-4	1.06	0.013	0.26	0.30	0.93	1.05	0.014	0.32	0.31	0.93

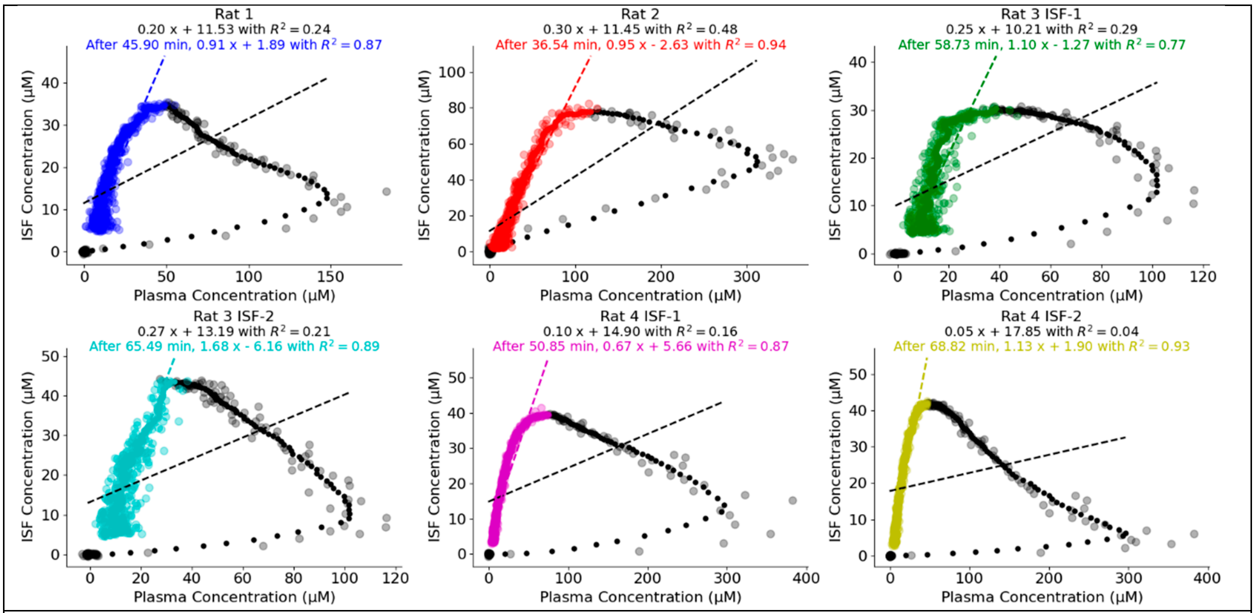

Figure S2. If we include the entire subcutaneous time course (i.e., including the extremely rapidly changing plasma drug concentrations seen before C_{max}; black dots), the correlation between plasma and ISF drug concentrations are relatively poor (the black dashed lines). If, instead, we only consider the period after the most rapid pharmacokinetic phases have largely equilibrated (i.e., after C_{max} has been achieved in the ISF), the correlation between plasma and ISF drug concentrations becomes quite strong (colored dashed lines). The individual plots shown here use the same color employed in **Figure 2 and 5**. The details of the linear fits can be found in **Table S3**.

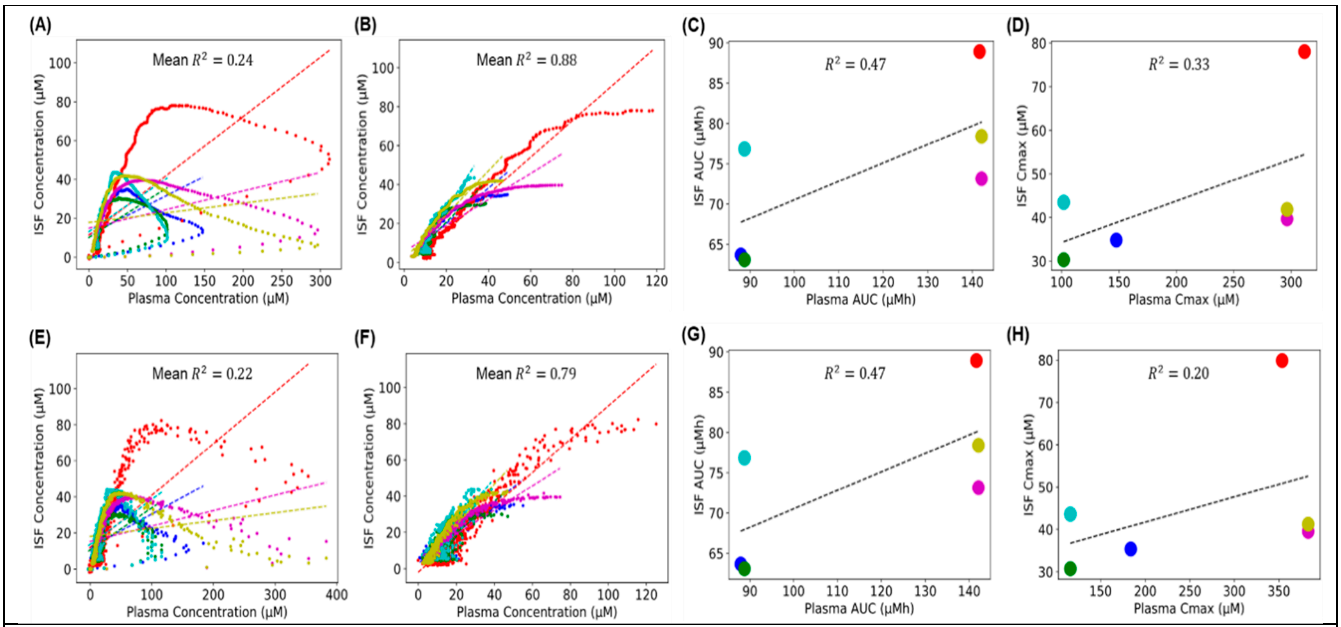

Figure S3. There is not much of a difference between using smoothed (top row) and "raw" data (bottom row) for the correlation analysis presented in **Figure 5**. The biggest difference is observed for C_{max} values, which is expected, as C_{max} is the most sensitive to smoothing operations. The detailed linear fit parameters can be found in **Table S3**.

Table S3. The slope and intercept values using smoothed vs raw concentration data, visualized in **Figure S2** and **S3**.

		Smoothed Data					Raw Data				
		Slope	Stand. error	Intercept	Stand. error	R^2	Slope	Stand. error	Intercept	Stand. error	R^2
All Data	Rat-1	0.20	0.014	11.53	0.46	0.24	0.19	0.013	11.83	0.46	0.23
	Rat-2	0.30	0.013	11.45	0.83	0.48	0.29	0.013	12.08	0.84	0.46
	Rat-3-1	0.25	0.018	10.21	0.57	0.29	0.23	0.018	10.62	0.56	0.27
	Rat-3-2	0.27	0.025	13.19	0.77	0.21	0.25	0.024	13.70	0.76	0.19
	Rat-4-1	0.10	0.010	14.90	0.66	0.16	0.09	0.010	15.26	0.65	0.15
	Rat-4-2	0.05	0.012	17.85	0.77	0.04	0.04	0.011	18.07	0.76	0.03
	Aver-age	0.20	0.015	13.19	0.68	0.24	0.18	0.015	13.59	0.67	0.22
After Cmax	Rat-1	0.91	0.015	1.89	0.28	0.87	0.84	0.018	2.98	0.34	0.80
	Rat-2	0.95	0.011	-2.63	0.36	0.94	0.92	0.013	-1.87	0.44	0.91
	Rat-3-1	1.10	0.033	-1.27	0.55	0.77	0.88	0.038	2.14	0.66	0.61
	Rat-3-2	1.68	0.032	-6.16	0.50	0.89	1.24	0.050	0.23	0.80	0.65
	Rat-4-1	0.67	0.014	5.66	0.35	0.87	0.67	0.014	5.70	0.36	0.86
	Rat-4-2	1.13	0.017	1.90	0.33	0.93	1.12	0.019	2.13	0.36	0.91
	Aver-age	1.07	0.020	-0.10	0.40	0.88	0.94	0.025	1.89	0.49	0.79
	C_{max}	0.10	0.068	24.75	15.60	0.33	0.06	0.060	29.82	17.07	0.20
	AUC	0.23	0.121	47.58	14.27	0.47	0.23	0.121	47.57	14.29	0.47

**Table S4.** Fitted plasma pharmacokinetic parameters.								
Parameter	Rat 1	Stand. error	Rat 2	Stand. error	Rat 3	Stand. error	Rat 4	Stand. error
k_E (min^{-1})	0.036	0.0008	0.050	0.0010	0.019	0.0009	0.057	0.0019
k_{AP} (min^{-1})	0.070	0.0037	0.059	0.0020	0.023	0.0011	0.080	0.0078
k_{PA} (min^{-1})	0.043	0.0025	0.030	0.0016	0.015	0.0019	0.068	0.0069
V_P (L)	0.138	0.0022	0.049	0.0006	0.111	0.0017	0.032	0.0008
RMSE (μM)	4.71		8.87		4.57		10.61	

To fit our blood drug concentration time courses, we use a two-compartment diffusion model described by the differential equations,

$$\frac{dC_P(t)}{dt} = k_{AP}\big(C_A(t) - C_P(t)\big) - k_E C_P(t) + \frac{1}{V_P}u(t)$$
$$\frac{dC_A(t)}{dt} = k_{PA}\big(C_P(t) - C_A(t)\big), \qquad \text{(Eq. S1)}$$

where $C_P(t)$ and $C_A(t)$ are concentrations in the plasma and in the abstract additional compartment at time t, respectively; $u(t)$ is the drug infusion rate at time t; k_E is the elimination rate of the drug from the plasma; k_{PA} is the rate constant capturing the impact of the plasma on to the abstract compartment, and k_{AP} is the vice versa; and V_P is the distribution volume of the plasma compartment.

Once the plasma time course is fitted to the (S1) with estimated parameter values, we take the estimated plasma time course as a set input value driving the ISF time courses. The ISF dynamics are then captured by three distinct models.

$$\frac{dC_{ISF}(t)}{dt} = k_{PI}\big(C_P(t) - C_{ISF}(t)\big) \qquad \text{(Eq. S2)}$$

$$\frac{dC_{ISF}(t)}{dt} = k_{IN}C_P(t) - k_{OUT}C_{ISF}(t) \qquad \text{(Eq. S3)}$$

$$\frac{dC_{ISF}(t)}{dt} = k_{PI}\big(C_P(t) - C_{ISF}(t)\big) - k_{DI}\big(C_D(t) - C_{ISF}(t)\big)$$
$$\frac{dC_D(t)}{dt} = k_{ID}\big(C_{ISF}(t) - C_D(t)\big), \qquad \text{(Eq. S4)}$$

Structural Model Identifiability

We note that all the models under consideration correspond to either a first order (Eq. S2 and S3) or a second order (Eq. S1 and S4) input–output relation. Indeed, we even fit our data using the transfer function descriptions first, since transfer functions (an algebraic frequency domain expression defining the input–output relation) uniquely describe the input–output relationship. Even so, the rate constants in equations (Eq. S1–S4) can be uniquely determined from the transfer function coefficients.

1. First Order Transfer Functions (Eq. S2 and S3)

We have a direct trivial correspondence between the rate constants and the transfer function coefficients in this case.

$$h_{diffusion}(s) = \frac{k_{PI}}{s + k_{PI}} \qquad \text{(Eq. S5)}$$

$$h_{differential}(s) = \frac{k_{IN}}{s + k_{OUT}} \qquad \text{(Eq. S6)}$$

where the transfer functions are obtained using the Laplace transform, and the rate constants (k_{PI}, k_{IN}, k_{OUT}) from equations (Eq. S2) and (Eq. S3) readily appear as transfer functions in (Eq. S5) and (Eq. S6).

2. Second Order Transfer Functions (Eq. S1 and S4)

The second order models are a bit more complicated than the first-order ones, but there is still a one-to-one correspondence between the transfer function coefficients and the rate constants.

a. Plasma Dynamics

Taking the Laplace transform of (Eq. S1), we reach to a transfer function from input ($u(t)$) to the plasma profile ($C_P(t)$).

$$h_P(s) = \frac{(s + k_{PA})/V_P}{s^2 + (k_E + k_{AP} + k_{PA})s + k_E k_{PA}} = \frac{\beta_1 s + \beta_0}{s^2 + \alpha_1 s + \alpha_0} \quad \text{(Eq. S7)}$$

The equivalent inverse transform from transfer function coefficients ($\alpha_1, \alpha_0, \beta_1, \beta_0$) to rate constants ($k_E, k_{AP}, k_{PA}, V_P$) can be done via the following relation;

$$\begin{aligned} V_P &= \frac{1}{\beta_1} \\ k_{PA} &= \frac{\beta_0}{\beta_1} \\ k_E &= \frac{\alpha_0 \beta_1}{\beta_0} \\ k_{AP} &= \alpha_1 - \frac{\alpha_0 \beta_1}{\beta_0} - \frac{\beta_0}{\beta_1} \end{aligned} \quad \text{(Eq. S8)}$$

The relationship (S8) is well defined, as long as $\beta_0 \neq 0$ and $\beta_1 \neq 0$. This is satisfied in our work, since transfer function coefficients are strictly positive if the rate constants are strictly positive, and because in our case of pharmacokinetic modeling, we already have a strict positivity requirement on all the rate constants.

The relationship (S8) is bijective for strictly positive rate constants (k_E, k_{AP}, k_{PA}, V_P). Suppose that for a given set of transfer function coefficients ($\alpha_1, \alpha_0, \beta_1, \beta_0$), we have two distinct sets of rate constants: (k_E, k_{AP}, k_{PA}, V_P) and (r_E, r_{AP}, r_{PA}, L_P). Then, using the relation (S8), we have

$$\begin{aligned} L_P &= \frac{1}{\beta_1} = V_P \\ r_{PA} &= \frac{\beta_0}{\beta_1} = \beta_0 L_P = \beta_0 V_P = k_{PA} \\ r_E &= \frac{\alpha_0 \beta_1}{\beta_0} = \frac{\alpha_0}{r_{PA}} = \frac{\alpha_0}{k_{PA}} = k_E \\ r_{AP} &= \alpha_1 - \frac{\alpha_0 \beta_1}{\beta_0} - \frac{\beta_0}{\beta_1} = \alpha_1 - r_E - r_{PA} = \alpha_1 - k_E - k_{PA} = k_{AP} \end{aligned} \quad \text{(Eq. S9)}$$

Therefore, (k_E, k_{AP}, k_{PA}, V_P) and (r_E, r_{AP}, r_{PA}, L_P) cannot be distinct. The reverse direction of any set of feasible rate constants (k_E, k_{AP}, k_{PA}, V_P) corresponds to a unique set of transfer functions ($\alpha_1, \alpha_0, \beta_1, \beta_0$), which can be shown in a similar and more trivial way. Therefore, the relation (S8) is a bijective map, meaning that the transfer function is identifiable.

Transfer function (S7) is a minimal order identifiable transfer function. This is to say that the numerator cannot be canceled from a root of the denominator. In order for this to happen, the root of the numerator ($s = -k_{PA}$) must also be a root of the denominator of (S7).

$$(-k_{PA})^2 + (k_E + k_{PA} + k_{AP})(-k_{PA}) + k_E k_{PA} = -k_{PA}k_{AP} = 0 \qquad \text{(Eq. S10)}$$

This means that we must either have $k_{PA} = 0$ or $k_{PA} = 0$ for the transfer function (S7) to be non-minimal, which is again out of scope for our work, as we enforce a strict positivity condition on our pharmacokinetic parameters.

In conclusion, as long as we operate under the condition of strictly positive rate constants, the rate constants are structurally identifiable because the corresponding transfer function is a minimal unique expression and the rate constants relate to the transfer function coefficients via bijective mapping.

b. Distal Compartment Model Dynamics

Next, we perform the same analysis for the distal compartment model (Eq. S4). This yields a transfer function

$$\mathrm{h_{diffusion}}(s) = \frac{k_{PI}s + k_{PI}k_{ID}}{s^2 + (k_{PI} + k_{ID} + k_{DI})s + k_{PI}k_{ID}} = \frac{\beta_1 s + \alpha_0}{s^2 + \alpha_1 s + \alpha_0} \qquad \text{(Eq. S11)}$$

Using a similar analysis to above, we have the following relationship between the transfer function coefficients $(\alpha_1, \alpha_0, \beta_1)$ and the rate constants (k_{PI}, k_{ID}, k_{DI})

$$\begin{aligned} k_{PI} &= \beta_1 \\ k_{ID} &= \frac{\alpha_0}{\beta_1} \\ k_{DI} &= \alpha_1 - \beta_1 - \frac{\alpha_0}{\beta_1} \end{aligned} \qquad \text{(Eq. S12)}$$

The relation (Eq. S11) is similarly well-defined, since we have $\beta_1 = k_{PI} > 0$. The relation (Eq. S11) is also similarly bijective from strictly positive (k_{PI}, k_{ID}, k_{DI}) to $(\alpha_1, \alpha_0, \beta_1)$. This can be seen by running the same contradiction proofs as above. Lastly, if we check for the algebraic irreducibility, we have the root of the numerator at $s = -k_{ID}$.

$$(-k_{ID})^2 + (k_{PI} + k_{ID} + k_{ID})(-k_{ID}) + k_{PI}k_{ID} = -k_{DI}k_{ID} = 0 \qquad \text{(Eq. S13)}$$

This again confirms that the transfer function (Eq. S10) is an algebraically irreducible transfer function for strictly positive rate constants (k_{PI}, k_{ID}, k_{DI}).

Table S5. ISF Modeling quality results for the parameters presented in **Table 1**.

Criteria	Model	Rat-1	Rat-2	Rat-3-1	Rat-3-2	Rat-4-1	Rat-4-2
BIC	Diffusion	4046.4	4813.3	2865.9	2226.6	3564.5	3459.6
	Differential	1856.2	2998.9	2183.4	2160.7	741.8	2336.4
	Distal	1862.7*	3005.3*	2784.4	2238.9*	750.6*	2342.6*

RMSE (μM)	Diffusion	5.07	14.83	5.34	2.68	10.92	9.76
	Differential	0.97	3.12	2.54	2.47	0.53	2.91
	Distal	0.97	3.12	4.83	2.68	0.53	2.91
Reciprocal Hessian Condition	Diffusion	1	1	1	1	1	1
	Differential	0.042	0.038	0.044	0.051	0.041	0.058
	Distal	1.17E-5	1.06E-8	1.55E-7	1.51E-6	1.59E-6	5.17E-7
*BIC values for models with near-zero estimated parameter values.							

Table S6. The approximated standard errors for ISF parameter estimations. Fits with near-boundary estimates are ignored.

	Rat-1	Rat-2	Rat-3-1	Rat-3-2	Rat-4-1	Rat-4-2
k_{PI}	0.0018	0.0035	0.0019	0.0025	0.0005	0.0006
k_{IN}	0.0018	0.0039	0.0020	0.0025	0.0009	0.0008
k_{OUT}	0.0027	0.0067	0.0029	0.0029	0.0020	0.0015
k_{PI}	N/A	N/A	0.7023	N/A	N/A	N/A
k_{DI}	N/A	N/A	4.7419	N/A	N/A	N/A
k_{ID}	N/A	N/A	0.0978	N/A	N/A	N/A

Table S7. Bootstrap results. Fits with near-boundary estimates are ignored.

	Rat-1			Rat-2			Rat-3-1		
	Original Estimate	Bootstrap		Original Estimate	Bootstrap		Original Estimate	Bootstrap	
		Mean	STD		Mean	STD		Mean	STD
k_{PI}	0.0239	0.0238	3.10E-04	0.0361	0.0361	4.64E-04	0.0231	0.0230	3.45E-04
k_{IN}	0.0258	0.0258	2.69E-04	0.0403	0.0403	3.98E-04	0.0245	0.0245	3.33E-04
k_{OUT}	0.0338	0.0338	4.60E-04	0.0620	0.0619	8.24E-04	0.0318	0.0317	5.43E-04
k_{PI}	0.0258	N/A	N/A	0.0403	N/A	N/A	0.1842	1.3477	0.547
k_{DI}	0.0080	N/A	N/A	0.0217	N/A	N/A	0.9601	0.0252	0.011
k_{ID}	5.08E-11	N/A	N/A	4.99E-12	N/A	N/A	0.1283	0.1923	0.064
	Rat-3-2			Rat-4-1			Rat-4-2		
	Original Estimate	Bootstrap		Original Estimate	Bootstrap		Original Estimate	Bootstrap	
		Mean	STD		Mean	STD		Mean	STD
k_{PI}	0.0313	0.0312	3.92E-04	0.0062	0.0062	2.28E-04	0.0080	0.0080	2.36E-04
k_{IN}	0.0313	0.0312	3.84E-04	0.0124	0.0124	1.82E-04	0.0115	0.0115	1.36E-04
k_{OUT}	0.0328	0.0328	5.11E-04	0.0225	0.0226	5.00E-04	0.0183	0.0184	3.63E-04
k_{PI}	0.0313	N/A	N/A	0.0124	N/A	N/A	0.0115	N/A	N/A
k_{DI}	3.24E-05	N/A	N/A	0.0102	N/A	N/A	0.0068	N/A	N/A
k_{ID}	2.9379	N/A	N/A	6.70E-05	N/A	N/A	1.20E-11	N/A	N/A
